# Supplementary material for: Comparative genomics of Tn6411 transposons carrying the blaIMP-1 gene in Pseudomonas aeruginosa
Source: PLoS One. 2024 Jul 9;19(7):e0306442. doi: 10.1371/journal.pone.0306442 (PMC11232968; doi:10.1371/journal.pone.0306442)
Supplement: Attachment — Submitted filename: Response to Reviewers.docx [file pone.0306442.s003.docx]

We are greatly appreciated to editor's contribution to this article. We have completed the modification of the paper format as required, and at the same time we have sought native English speakers to help us polish the language in this article. We have also released the relevant sequences in GenBank for readers' reference.

Reviewer #1: The manuscript by Wang and cols. analyzes the genetic detection of transposon Tn*6411* flanking the *bla*_IMP-1_ carbapenem-resistant gene in two *Pseudomonas aeruginosa* of clinical origin. This manuscript is relevant because it reports the evolution and potential dissemination of Tn*6411* in clones of *P. aeruginosa* not typically reported, considered not pandemic, which highlights its role in spreading last-resort antibiotics. However, I have some recommendations that could improve the manuscript:

Response: Thank Reviewer 1;

we learned a lot from the valuable advice and suggestions. We have thoroughly revised our manuscript. The issues have been addressed point-by-point. Please see our responses below.

**1. The quality of the two figures must be improved.**

Response: Revised (**Fig 1 and Fig 2**);

The clarity of the image is directly correlated with the reader's perception. Currently, we have enhanced the picture's clarity using photoshop software by increasing the original 300 dpi to 600 dpi and converting the output from “png” to “tif” format. As a result, there has been a significant improvement in clarity.

**2. Line 81. Please include the version (year) of the CLSI guidelines.**

Response: Added (**Line 84-86**): “**Drug resistance and sensitivity were judged based on the Clinical and Laboratory Standards Institute guidelines (2019)**.”

Thank you for the expert's reminder. We overlooked this aspect, as CLSI undergoes minor changes with each update, so it is important to specify the version we are referring to. Therefore, we have added the CLSI version (2019) for reader reference.

**3. Line 109-111. Why did the authors not include the OD600 as a normalization tool for the number of cells in the conjugation experiments? Why was a *P. aeruginosa* strain not used as a recipient instead of an *E. coli*? Please add more information about it in the discussion section.**

Response: Added (**Line 138-139**): “**The flat colony counting method was used to ensure an equal 1:1 mixture of each strain with *E. coli* J53.**”

The description of "equal volume" in this article is overly general, and a normalization method should be employed to describe. The OD600 method is used to estimate the number of cells by measuring the optical density of the cell suspension at a wavelength of 600 nanometers. However, it lacks the ability to differentiate between living and dead cells, and variations in light absorption properties among different cell types or within the same cell at different growth stages, which can lead to inaccuracies in OD600 measurements. This limitation is particularly significant in experiments requiring precise cell counting. Therefore, this study employed the technique of flat colony counting method to ascertain if the quantities were in an equal ratio. Currently, this aspect of the experimental method has been refined.

There were several uncertainties regarding the experimental conditions when using non-*E. coli* J53 as recipient cells for conjugation. Therefore, the *E. coli* J53 strain, which is commonly used for conjugation experiments, was chosen as the recipient strain in this study.

**4. Line 150-151. Why was strain 18083286 included in the conjugation experiments even though "no plasmid was detected"? Maybe the order of the experiments should be revised.**

Response:

In this study, we initially identified high similarity between the nucleic acid sequences of strain 18083286 and 18081308 using next-generation sequencing. Consequently, only strain 18083286 was randomly sequenced for the single molecule real-time sequencing. Despite not detecting plasmids in the bacterium through sequencing, it was important to note that current sequencing technology may easily overlook low-copy number plasmids. Therefore, the presence of bacteria carrying low-copy plasmids cannot be ruled out by sequencing alone.

Comparison with the GenBank database revealed that Tn*6411* sequences existed not only in *Pseudomonas aeruginosa* but also in *Acinetobacter* spp.. Subsequently, conjugation experiments were conducted to ascertain whether the *bla*_IMP-1_ gene in Tn*6411* is transferable. Hence, we performed conjugation experiments after sequencing to validate the transferability of Tn*6411*-*bla*_IMP-1_.

Reviewer #2: The paper “Potential transferability and evolution of Tn*6411* transposons carrying the *bla*_IMP-1_ gene in *Pseudomonas aeruginosa*” is interesting. Describes a transposon that allows the mobility of resistance genes such as *bla*_IMP_. However, major corrections must be made to make it clear.

Response: Thank Reviewer 2’s suggestions.

We have revised our manuscript point-by-point. Please see the following response for details:

**1. You must change Figure 1 because it cannot be seen.**

Response: Revised (**Fig 1 and Fig 2**);

The clarity of the image was directly related to how the reader seen it. We recently improved the picture's clarity using photoshop software, increasing the original 300 dpi to 600 dpi and changing the output from “png” to “tif” format. As a result, there had been a significant improvement in clarity.

**2. Table 1 only describes the resistance profile of strain 18083286. If strain 18081308 has the same resistance profile, it should be said in the footer of the table or the title, and if not, the resistance profile of this strain should also be included.**

Response: Revised (**Line 153-155**): “**Table 1 shows the drug resistance spectrum of strain 18083286, which was consistent with that of strain 18081308.**”

Thank you once again for the expert's reminder. The drug resistance information of strains 18083286 and 18081308 is indicated consistent in the article (Line 174), but only the drug resistance information of strain 18083286 is included in the Table 1. This omission may lead to confusion among some readers. As suggested by experts, when referring to Table 1, it is explained that the resistance of the two is consistent.

**3. The authors describe that this transposon carrying *bla*_IMP-1_ is found in one of the strains on the chromosome and in the other on a plasmid. However, they do not describe the plasmid carrying the transposon by sequencing, which would be important to see if it coincides with the results obtained in the assay of the conjugation.**

Response:

The strain Tn*6411*_18083286_ identified in this study is chromosomally located, while the plasmid-mediated Tn*6411*-like_p201330-IMP_, Tn*6411*-like_pE47_, Tn*6411*_pWM98B_, and Tn*6411*_pMKPA34-2_ are carried by the selected strains in GenBank (**Line 190-191**). Whether conjugal transfer events can occur has not been reported.

**4. In methods and results, the authors do not specify the strain used. The article, in general, describes one of the strains, and the question remains whether the other strain had the same thing or if there are changes, so it should include both strains.**

Response: Revised (**Line 79-139; Line 153-155**);

The absence of strain labeling poses a challenge for readers in comprehending this article. Currently, we have provided specific details about the strains used to enhance readers' understanding of this study.

The nucleotide identity between strains 18083286 and 18081308 was evaluated using ANI. Due to the discontinuity and incompleteness of the next-generation sequencing (NGS) results, there was an interference effect on the analysis of nucleobase absences. In this study, strain 18083286 was randomly selected to undergo another DNA extraction step, and the newly obtained DNA was subsequently single molecule real-time (SMRT) sequenced using a PacBio RSII sequencer. Therefore, the phenotype experiment in this paper was analyzed between strains 18083286 and 18083286. Subsequent sequence analysis was carried out between strain 18083286 and the GenBank database.

**5. The authors show that two methodologies were used for sequencing strain 18083286; however, they do not say if it made a hybrid assembly of the sequences**.

Response: Revised (**Line 94-128**)

We initially performed next-generation sequencing on 18083286 to preliminarily assess its nucleic acid sequence identity with strain 18081308. Due to the high-level similarity of nucleic acid sequence, presence of drug-resistance genes and phenotypes between 18083286 and 18081308, we proceeded single molecule real-time sequencing on a randomly selected strain. As a result, both next-generation and real-time sequencing of this bacterium were conducted as non-mixed sequencing. The experimental methods of next-generation sequencing and single molecule real-time sequencing analysis have been described separately to ensure clear expression.
