# Supplementary material for: Comparative genomics of Tn6411 transposons carrying the blaIMP-1 gene in Pseudomonas aeruginosa
Source: PLoS One. 2024 Jul 9;19(7):e0306442. doi: 10.1371/journal.pone.0306442 (PMC11232968; doi:10.1371/journal.pone.0306442)
Supplement: Attachment — Submitted filename: Response to Reviewers.docx [file pone.0306442.s004.docx]

**Reviewer #1**

**1. I partially agree that in this case, "the clarity of the image is directly correlated with the reader's perception". However, once the figures meet the standards indicated in the guide for authors, it should be fine, and the production team will say something in case not.**

Thank you for reviewer’s understanding. The figures have been adjusted based on “The guide for authors”, and it is possible that the journal may have compressed the figures when storing them. If the production team needs them in the future, we will be happy to provide assistance, including supplying the original figures.

**2.** **Regarding the conjugation experiments, it is not clear to me why you wanted to validate the transferability of Tn*6411*-*bla*_IMP-1_ by conjugation in a plasmid-free strain. Why don't you evaluate this transferability by transformation instead? which could provide more comprehensive results.**

Thank you for reviewer’s advice. Donor DNA and competent bacterial cells were needed for transformation instead. However, the strain 18083286 in this study did not contain plasmids, which made it challenging to extract DNA from the Tn*6411* target fragment. The process of conducting adsorption experiments for free DNA without a plasmid-carrier has not been mature yet, adding to the uncertainty of the experiment. Therefore, this study didn’t conduct any transformation experiments.

We can also determine that it is only capable of vertical transmission on the chromosome by single molecule real-time (SMRT) sequencing and analyzing the structure of Tn*6411*. Therefore, the conjugation experiment in this study was not very meaningful and will be removed.

**Reviewer #2**

**1. The authors do not answer whether they carried out a hybrid assembly for strain 18083286 because they carried out two sequencing by two different methods. In that case, they could carry out a hybrid assembly that would provide them with more information. They could be surer of your information. I suggest that it be carried out or clarify why it was carried out by two sequencing methodologies, both in methods and results.**

Thank you for the reviewer’s advice. The reasons for conducting next generation sequencing (NGS) of the strains in this study are as follows:

(1) Due to the discontinuity and incompleteness of the NGS results, there was an interference effect on the analysis of nucleobase absences. To better understand the sequence structure, we conducted sequencing across single molecule real-time (SMRT) sequencing (Line: 106-110). Before proceeding with SMRT sequencing, in order to consider the cost, we initially used next generation sequencing to screen the strains;

(2) For the SMRT sequencing assembly, we utilized NGS calibration (Line: 110-116).

**2. Line 222. The authors describe the Tn*6411* transposon through bioinformatic analysis and compare it with others reported in GenBank. However, they cannot say that they determined the evolution of this transposon since, with this analysis, they can only say that there are changes. Other studies must be done to know evolution, so the word evolution must be eliminated from the title.**

Thank you for the reviewer’s reminder. The title has been modified to “Comparative genomics of Tn*6411* transposons carrying the *bla*_IMP-1_ gene in *Pseudomonas aeruginosa*”.

**3. Line 198. The authors say that the observed modifications make Tn*6411* and Tn*6411*-like stable, which must be demonstrated experimentally. Like in line 229, they should remove “forming a stable structure when you capture the type1 integron with *bla*_IMP-1_ because bioinformatic analysis cannot determine."**

Thank you once again for the reviewer’s reminder. The bioinformatics focused on analyzing and predicting the function and structure of sequence, including mobile genetic elements (like integrons and transposons). According to the sequence perspective, the deletion of transposon unit sequences in this study could result in the mobility loss of Tn*6411* and integrons (Line: 209-211). However, reviewer has emphasized that further experimental verification was needed to confirm whether they were stably present. Therefore, we removed speculations that have not been verified by experiments.

**4. Line 116. It is unclear why a conjugation test should be performed on strain 18083286, given that the bioinformatic analysis showed that Tn*6411* with *bla*_IMP-1_ is located on the chromosome. We know that it will only be transferred vertically.**

We also thought that using single molecule real-time (SMRT) sequencing to analyze the structure of Tn*6411* will show that it can only be passed down vertically on the chromosome. Therefore, the conjugation experiment in this study wasn't very useful and will be eliminated.

**5. The authors say that the plasmid cannot be detected because they have a low copy number; however, it is not a factor for not observing it in the type of sequencing they carry out because, in these cases, libraries are created that in some way would help with this problem. On the other hand, they detect it in strain 18081308. However, they do not describe it, at least what refers to its conjugation machinery, to relate it to what was found in the conjugation assay. So, they must change their conclusion (line 233).**

Thank you for reviewer’s clear explanation.

Since strain 18081308 only underwent NGS, we were unable to determine whether it carried plasmids, so it was not mentioned in this study.

We have updated the conclusion by adjusting speculative statements about gene transfer that cannot be confirmed through bioinformatics analysis in this study (Line: 241-259).

**6. I continue to insist that the figures are of very poor quality and do not look properly. These must be clear to any reader.**

The figures have been adjusted according to "The guide for authors", and it's possible that the journal may have shrunk the figures when saving them. If the production team needs them in the future, we'd be happy to help, including providing the original figures.

**7. The authors give the title “Potential transferability and evolution”; however, the only thing they do is a description of the transposon found in their strains and a comparison with other transposons reported in GenBank, so they cannot talk about evolution or Potential transferability. Only with a bioinformatic analysis, in addition to this not being adequately documented in the article**

To better align with the research focus of this article, the title has been modified to “Comparative genomics of Tn*6411* transposons carrying the *bla*_IMP-1_ gene in *Pseudomonas aeruginosa*”.
